# Photoperiodic Remodeling of Adiposity and Energy Metabolism in Non-Human Mammals

**DOI:** 10.3390/ijms24021008

**Published:** 2023-01-05

**Authors:** Èlia Navarro-Masip, Alexandre Caron, Miquel Mulero, Lluís Arola, Gerard Aragonès

**Affiliations:** 1 Nutrigenomics Research Group, Department of Biochemistry and Biotechnology, Universitat Rovira i Virgili, 43007 Tarragona, Spain; 2 Faculty of Pharmacy, Université Laval, Québec City, QC G1V 0A6, Canada

**Keywords:** adipose tissue, leptin, melatonin, obesity, photoperiod, seasonality

## Abstract

Energy homeostasis and metabolism in mammals are strongly influenced by seasonal changes. Variations in photoperiod patterns drive adaptations in body weight and adiposity, reflecting changes in the regulation of food intake and energy expenditure. Humans also show distinct patterns of energy balance depending on the season, being more susceptible to gaining weight during a specific time of the year. Changes in body weight are mainly reflected by the adipose tissue, which is a key metabolic tissue and is highly affected by circannual rhythms. Mostly, in summer-like (long-active) photoperiod, adipocytes adopt a rather anabolic profile, more predisposed to store energy, while food intake increases and energy expenditure is reduced. These metabolic adaptations involve molecular modifications, some of which have been studied during the last years and are summarized in this review. In addition, there is a bidirectional relation between obesity and the seasonal responses, with obesity disrupting some of the seasonal responses observed in healthy mammals, and altered seasonality being highly associated with increased risk of developing obesity. This suggests that changes in photoperiod produce important metabolic alterations in healthy organisms. Biological rhythms impact the regulation of metabolism to different extents, some of which are already known, but further research is needed to fully understand the relationship between energy balance and seasonality.

## 1. Introduction

Life on Earth has substantially evolved following seasonal cyclic changes, reaching the times in which we are living today. Variations in temperature, food availability or daylength are some of the environmental signals that all organisms periodically consider adapting to in advance of upcoming environmental changes. Being able to adapt to the changing environment provides survival advantages to most living organisms, as each period of the year requires different adjustments. These adjustments can take a few weeks to months to be established and include behavioral, morphological and physiological variations [1]. This highlights the importance of properly sensing circannual changes in nature.

Mammalian control of energy metabolism is extremely complex and is also clearly regulated by circannual changes [2]. For instance, the photoperiod is the strongest seasonal cue used for mammals to synchronize their energy balance with the environment [1]. In fact, a close relationship between photoperiod and body weight has been observed in two recent studies in humans, both revealing different patterns of weight gain during different photoperiods [3,4]. In general, although mammals can respond to long photoperiod (LP) or short photoperiod (SP) differently [5], all of them show seasonal changes in body weight and adiposity [6]. In fact, the amount of fat stores is optimized in mammals to be metabolically prepared for the forthcoming conditions. As a general rule, the anabolic-growth phase is usually timed to LP, when food is abundant, and the reciprocal catabolic-anorexigenic phase is timed to SP, when food is scarce [7].

In addition, a conserved neuroendocrine pathway across mammals that involves photoperiod sensing exists [2]. Nevertheless, the molecular mechanisms that shape the specific metabolic responses to photoperiod are barely described. Thereby, the aim of this review is to summarize the current knowledge on the biological strategies involved in the photoperiodic remodeling of energy metabolism in order to provide potential therapeutic strategies for the management of body weight and adiposity in a similar way to what happens during seasonal cyclic changes. To note, due to the lack of data on human studies related to this topic, we have decided to focus on animal-based studies, especially rodent-based studies, which have been widely used to understand the biological and molecular changes that occur in different seasons.

## 2. Melatonin Signaling and Calendar Cells

In mammals, changes in the light/dark cycle are sensed with the melanopsin-containing retinal ganglion cells which signal the information to some 10,000–15,000 neurons located in the suprachiasmatic nucleus (SCN) of the hypothalamus [5]. These neurons then send extensive connections to the paraventricular nucleus (PVN) of the hypothalamus, which can integrate this information or relay it to the superior cervical ganglion located in the sympathetic chain. Sympathetic fibers originating in the superior cervical ganglion project to the pineal gland, ultimately allowing the perception of photoperiod by pinealocytes. This information is transduced into an endocrine signal in the form of a rhythmic secretion of melatonin into the blood stream. The synthesis of melatonin results from the regulation of the pineal gland using the endogenous circadian oscillator that is entrained to the light/dark cycle [6]. Concentrations of melatonin in both the blood and pineal gland are low during the light phase and peak at night. The duration of melatonin secretion is therefore directly associated with the duration of the night and is used to drive photoperiodic responses. Thus, melatonin secretion is greater in SP compared with LP.

The *Pars Tuberalis* (PT) of the anterior pituitary gland is one of the main sites of action of melatonin, and represents the key site for regulation of seasonal rhythms by this hormone. The PT contains cells expressing high affinity melatonin 1 receptors (MT1 or MTNR1A) [8]. These specialized melatonin sensitive cells are known as “*calendar cells*”, due to their ability to sense the duration of melatonin exposure (that decodes daylength) and to generate long-term physiological responses [8]. A recent study performed in a ram of the Soay breed of sheep demonstrated that an intact PT is required to exert the photoperiodic control of seasonal metabolic cycles including appetite and body weight regulation [7]. Several studies have demonstrated that an injection of melatonin during a maintained long-day photoperiod mimicked the response to a short-day photoperiod, confirming the main endocrine function of melatonin in coordinating timing of seasonal physiology [9,10,11].

**Figure 1:**
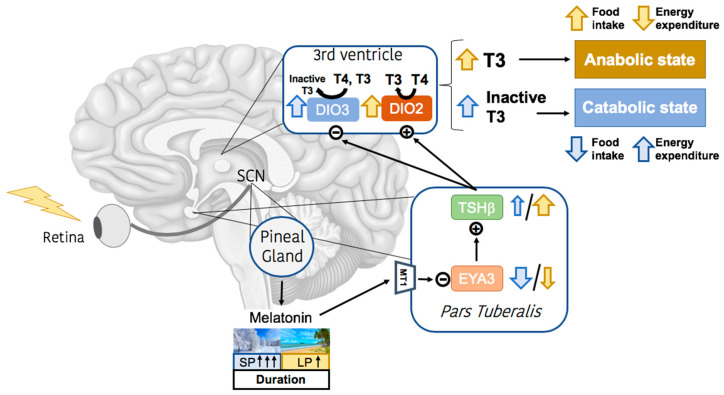
Melatonin seasonal signaling in the hypothalamus. In mammals, a luminous signal is received by melanopsin-containing cells in the retina, and is transferred to the SCN in the hypothalamus. Pinealocytes receive the information via SNS and respond by synthesizing and secreting melatonin. MT1 receptors in the “*calendar cells*” of the *Pars Tuberalis* detect melatonin signal, that acts by inhibiting EYA3 function, and therefore blocking TSHβ secretion. Depending on the duration of melatonin signaling, the inhibition of this pathway is stronger (during the SP or winter) or lighter (during the LP or summer). In summer-like photoperiod, TSHβ is released and acts by inhibiting DIO3 and stimulating DIO2 function in tanycytes. DIO2 induces T3 production and favors a rather anabolic state in the CNS, increasing food intake and reducing energy expenditure. In the winter-like photoperiod, TSHβ is not released by tanycytes and DIO3 increases its functionality, converting T4 into inactive metabolites of T3, favoring a rather catabolic state by reducing food intake and increasing energy expenditure. Abbreviations: CNS: Central Nervous System; DIO2: *Deiodinase 2*; DIO3: *Deiodinase 3*; EYA3: Eyes absent 3; LP: Long Photoperiod; SCN: Suprachiasmatic Nucleus; SNS: Sympathetic Nervous System; SP: Short Photoperiod; T3: Triiodothyronine; T4: Thyroxine; TSHβ: Thyroid Stimulating Hormone β Subunit.

As shown in Figure 1, melatonin signaling negatively regulates the expression of *Eyes absent 3* (EYA3) in the calendar cells of the PT, which in turn stimulates the release of *Thyroid stimulating hormone β subunit* (TSHβ). TSHβ then binds to its receptors located on tanycytes, which are specialized glial cells located in the ependymal lining of the third ventricle of the hypothalamus [12,13]. TSHβ exerts its actions by inducing the *Deiodinase 2* (DIO2) expression converting thyroxine (T4) into triiodothyronine (T3), which has a direct role in regulating appetite and energy homeostasis [12,14]. As nocturnal duration of melatonin is shorter in LP compared with SP, TSHβ secretion is not downregulated during LP and the resulting increase in T3 promotes anabolic responses. In contrast, the long duration of melatonin peak during SP reduces TSHβ secretion and, subsequently, the expression of *Deiodinase 3* (DIO3) is significantly upregulated and T4 is rapidly converted into its inactive (reverse T3 (rT3)) form, favoring catabolic actions [5]. Considering the widely established catabolic role of peripheral T3 in the organism, it may appear controversial that the effects of T3 in the central nervous system are anabolic. However, studies on thyroid hormones performed in recent years have extended and confirmed that T3 acts through many pathways and exerts different functions within the hypothalamus. The most convincing hypothesis suggests that the seasonal effects on central thyroid hormones are different from the conventional peripheral control of the hypothalmo-hypophyso-thyroid axis [5,15].

## 3. Effects of Photoperiod on White Adipose Tissue Metabolism

To understand the molecular mechanisms that drive body weight changes, many animal studies have been performed to establish the impact of photoperiod on adiposity and fat mass. Most studies were performed in rodents, including Siberian Hamsters (*Phodopus sungorus*) and Fischer 344 (F344) rats, which are proposed to be the most appropriate models as they exhibit important responses to changes in photoperiod [16,17,18]. The main conclusions from these models are that SP induces a significant reduction of subcutaneous and visceral white adipose tissue (WAT) depots without affecting food intake [16,17,18]. Similarly, decreased adiposity was also observed in C57BL/6 mice and obese Zucker rats exposed to shortened light exposure [19], confirming a pivotal role of WAT plasticity in response to photoperiod changes.

### 3.1. Metabolic Regulation of White Adipose Tissue

WAT represents an organ specialized in storing energy. It is composed of several cell types, with the adipocytes being the most abundant. These cells have a unilocular lipid droplet, few mitochondria and low oxidative rate. WAT has a high capacity for storing energy in the form of triglycerides (TG) and is responsible for maintaining the energy balance of an organism through accumulating or releasing energy in the form of fatty acids (FA). Interestingly, WAT depots are distributed throughout the body, showing different metabolic properties depending on their locations. The subcutaneous WAT (scWAT) is a rather protective tissue, with smaller, healthier adipocytes that are metabolically active and help prevent the deposition of TG in non-adipose organs. An example of scWAT is the inguinal WAT (iWAT), located in the groins in rodents. On the other hand, visceral WAT (vWAT) depots include mesenteric, gonadal, epicardial, retroperitoneal, omental, peri-renal and epidydimal depots. When in excess, these depots promote a rather unhealthy profile compared with scWAT. The vWAT contains larger and likely dysfunctional adipocytes and favors a pro-inflammatory state. In fact, the development of visceral depots in excess is strongly related to metabolic disorders and represents an independent predictor of mortality in humans [20].

**Figure 2:**
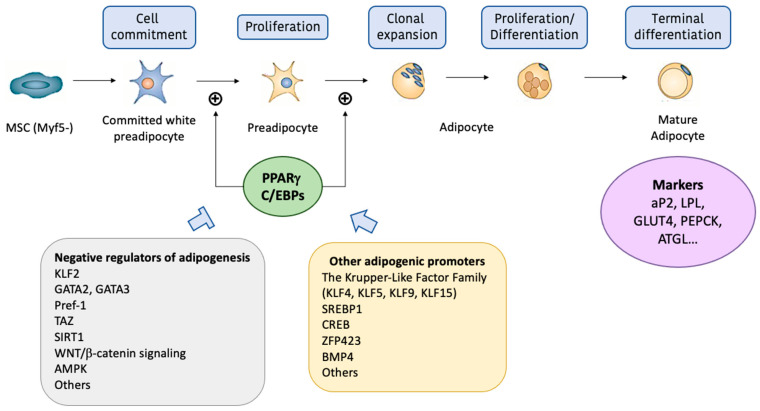
Schematic representation of adipogenesis and its molecular regulation. Non-expressing Myf5 mesenchymal stem cell (MSC) transitions to committed white preadipocyte, where adipogenic stimuli allows its proliferation through the activation of pro-adipogenic transcription factors, such as *peroxisome proliferator-activated receptor γ* (PPARγ) and some members of *CCAAT/enhancer binding protein family* (C/EBPs). Then, the preadipocyte goes through clonal expansion, which is the beginning of its differentiation and is also enhanced by PPARγ and C/EBPs, among others. During the differentiation phase, the adipocyte shows its typical morphological characteristics, such as a high amount of fat content, an eccentric nucleus and low content of mitochondria. The final step is the terminal differentiation, where the adipocyte is already mature and functional. During the early and mature differentiation, adipocytes express specific markers such as *Adipocyte fatty acid binding protein 2* (aP2), *glucose transporter 4* (GLUT4), *lipoprotein lipase* (LPL), *phosphoenol pyruvate carboxykinase* (PEPK) or *adipocyte triglyceride lipase* (ATGL). Adipogenesis is negatively regulated by some molecules and/or signaling pathways such as shown on the left side of the figure. Contrarily, positive regulators allow the development of this process (shown on the right side). Other abbreviations: AMPK: Adenosine Monophosphate-Activated Protein Kinase; BMP4: Bone morphogenic protein 4; CREB: Cyclic AMP Response Element-Binding Protein; Pref-1: Preadipocyte Factor 1; SIRT1: Histone Deacetylase Sirtuin 1; SREBP1: Sterol Regulatory Element-Binding Protein 1; TAZ: Transcriptional-Coactivator with PDZ-Binding Motif; ZFP423: Zinc Finger Protein 423.

WAT develops through a process called adipogenesis, which is strongly regulated by a complex network of transcription factors (Figure 2). Adipogenesis is a two-step developmental process in which multipotent Myf5-mesenchymal stem cells (MSCs) within the stroma differentiate to preadipocytes, which then undergo a secondary differentiation step and become lipid-filled adipocytes [21]. The first step of adipogenesis is the transition of embryonic stem cells to MSCs, which then proliferate to committed white preadipocytes [22]. These preadipocytes can then become mature white adipocytes by the addition of adipogenic stimuli, such as glucocorticoids, insulin or cyclic adenosine monophosphate (cAMP) [23]. The transcription factors that mainly drive adipogenesis are *Peroxisome proliferator-activated receptor γ* (PPARγ) and the family of *CCAAT/enhancer binding proteins* (C/EBPs), with C/EBPα suggested to be the most important. PPARγ is a nuclear receptor which acts as the master regulator of adipogenesis by modulating the initial phases [24] and maintaining the adipocyte lineage differentiation [25]. The C/EBP family of transcription factors interacts with the CCAAT box, which is a short sequence of nucleotides present in several gene promoters involved in the activation of transcription [26]. PPARγ and C/EBPs are intimately linked, with PPARγ promoting C/EBPs expression, and C/EBPs enhancing PPARγ expression and inducing the expression of other pro-adipogenic factors [27]. The most relevant genes that appear during the late stage of adipogenesis are *Adipocyte fatty acid binding protein 2* (aP2), *Lipoprotein lipase* (LPL), *Glucose transporter 4* (GLUT4) and *Phosphoenol pyruvate carboxykinase* (PEPCK). Overall, PPARγ and C/EBPα represent two key molecules that work in synchrony to initiate and regulate preadipocyte differentiation into mature adipocytes, by reacting to adipogenic stimuli [28]. On the other hand, negative regulators of adipocyte differentiation include the *Wnt/β-catenin* signaling pathway [29] and the protein *Adenosine monophosphate (AMP)-activated protein kinase* (AMPK), which acts as an energy sensor to regulate energy homeostasis [30]. Other molecules that positively and negatively control adipogenesis are shown in Figure 2.

**Figure 3:**
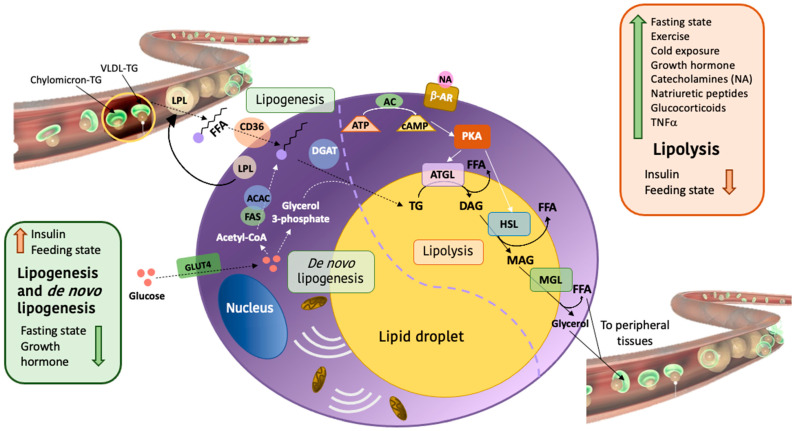
Regulation of lipid metabolism in adipocytes. The presence of insulin and/or a feeding state enhances the process of lipogenesis and de novo lipogenesis (marked by dashed arrows). On the one hand, the adipocytes obtain FFA (free fatty acids) through the LPL (*lipoprotein lipase*)-mediated breakdown of TG (triglycerides) from its circulating transporters. These FFA enter the adipocytes through the CD36 (*cluster of differentiation 36*) membrane transporter and are again converted into TG, by DGAT (*diacylglycerol acyltransferase*). On the other hand, adipocytes can convert glucose into FFA via de novo lipogenesis. This process is inhibited by a fasting state or the growth hormone which, together with many other cues, activate lipolysis (marked by solid arrows). In this case, the increase of cAMP activates the PKA (*protein kinase A*) function, and that promotes the activity of lipolytic enzymes. Hence, TG are converted into Glycerol and FFA, that go into circulation and are used by peripheral tissues that need energy. Other abbreviations: β-AR: β-adrenergic receptor; AC: *adenylyl cyclase*; ACAC: *acetyl-CoA carboxylase 1*; ATGL: *adipocyte triglyceride lipase*; DAG: diacylglycerol; FAS: *fatty acid synthase*; HSL: *hormone sensitive lipase*; MAG: monoacylglycerol; MGL: *monoacylglycerol lipase*; NA: noradrenaline; VLDL-TG: triglyceride containing-very low-density lipoprotein.

WAT stores energy through lipogenesis and de novo lipogenesis, two processes that are enhanced in the postprandial state when insulin levels increase (Figure 3). Adipocytes can accumulate lipids from circulation by re-esterifying free FA (FFA) that have been liberated from circulating TG through the action of LPL. Briefly, FFA are transported into the adipocytes through specialized transporters such as CD36 and converted into TG for storage. On the other hand, de novo lipogenesis is induced following insulin-dependent translocation of GLUT4 receptors to the adipocyte’s membrane, which increases glucose uptake [31]. Through glycolysis, glucose is then converted into pyruvate, which is converted to acetyl-CoA by the enzyme *Pyruvate dehydrogenase* (PDH) [32]. Acetyl-CoA can lead to the synthesis of FA through the action of *Acetyl-CoA carboxylase 1* (ACAC) and *Fatty acid synthase* (FAS). The opposite process, called lipolysis, allows the adipocytes to generate energy and release FFA when needed. This pathway is activated by increased cAMP levels following diverse stimuli such as fasting, exercise, cold exposure or catecholamines secretion. Lipolysis involves the action of different enzymes including *Protein kinase A* (PKA), *Adipose triglyceride lipase* (ATGL) and *Hormone sensitive lipase* (HSL) (Figure 3). These enzymes drive the breakdown of TG and its conversion into FFA and glycerol, which are then released into the blood stream and used by other tissues that require energy [33,34].

Interestingly, it is possible to stimulate the development of brite (brown in white) or beige cells in WAT (browning), following cold exposure or treatment with beta-adrenergic agonists [35]. Similar to brown adipocytes, which will be further explained in this review, brite or beige adipocytes contain *Uncoupling protein 1* (UCP1) in their mitochondria, which contributes to an increase in the mitochondrial oxidation [36]. However, the contribution of these cells to increasing global metabolic oxidation has been shown to be low [37]. Some of the brite cells arise from progenitors expressing *Transmembrane Protein 26* (TMEM26) and the prototype of costimulatory molecules for T cells CD137, or can be derived from smooth muscle cells. Although the origin of all beige cells is still unknown, it has been suggested that beige cells could arise not only through differentiation of precursor cells under certain stimuli, but also by transdifferentiation of pre-existing white adipocytes [35]. Its activity is protective against inflammation and metabolic diseases caused by obesity; therefore, to stimulate the activity and/or the differentiation of brown and beige adipocytes is increasingly considered an encouraging option for obesity treatment [38] or, at least, for protecting against its adverse metabolic consequences [39].

Furthermore, WAT acts as a real endocrine organ, secreting a wide range of hormones, known as adipokines, to regulate several physiological processes affecting the metabolic profile, such as food intake, glucose homeostasis and inflammation. The most characteristic adipokines are leptin, which is typically secreted in proportion to the amount of energy stored in WAT, and adiponectin, which levels are associated with a metabolic healthy state [25]. As an important endocrine organ, WAT is responsible for many metabolic functions that affect the whole organism.

### 3.2. Photoperiodic Remodeling of WAT Metabolism

WAT appears as a critical tissue at different levels, associated with positive outcomes when functionally and metabolically healthy. However, alterations in WAT physiology favor a general pro-inflammatory state [40]. Therefore, any situation that might influence the metabolic regulation of WAT can provide valuable information about the molecular pathways that modulate its functionality. In this context, the seasonal cyclic changes in WAT could provide potential strategies for the regulation of WAT metabolism in a similar manner to what happens during seasonal adaptations.

Table 1 summarizes the most relevant studies in different mammal species that have focused on exploring molecular changes of WAT metabolism in response to different photoperiod regimes, even when no significant changes in total body weight were observed. For instance, an increase in the adipocyte number and a decrease in the adipocyte size was reported in vWAT of F344 rats exposed to SP. At the molecular level, the adipogenesis and lipogenesis pathways in this tissue were reduced in SP through the downregulation of *Pparγ, C/ebpα* and *Fasn* [18]. To a similar extent, adipogenesis was also reduced in scWAT of *Psammomys obesus* rats exposed to SP [41]. Interestingly, a higher lipolytic activity was described in different WAT depots of Siberian Hamsters housed under SP [9], and the lipogenic metabolism was clearly downregulated in the epididymal WAT of Brandt’s voles exposed to SP [42] although no differences were reported in field voles exposed to the same conditions [43]. In addition, decreased glucose transport in perivascular WAT was reported in C57BL/6 mice housed under prolonged light exposure [44], indicating that the functionality of WAT is affected at different extents when mice are exposed to distinct photoperiod conditions. Moreover, adipose browning was also regulated by photoperiod in some types of WAT such as inguinal, retroperitoneal and epididymal. Accordingly, higher expression levels of *Ucp1* and *Prdm16* were observed in retroperitoneal WAT of F344 rats housed under LP [18], and *Pgc-1α* expression levels were reduced in the scWAT of *Psammomys obesus* rats during SP [41].

## 4. Effects of Photoperiod on Food Intake and Nutrient Choice

Seasonal changes in WAT usually go together with decreased food intake [45,46,47,48]. In fact, many studies have reported that lowering energy intake is needed for F344 rats to reduce body weight under SP, as rats that did not reduce energy intake did not show any differences in body weight during this photoperiod [18,49]. However, other studies found no significant differences in food intake between groups housed under different photoperiods [41,50,51], indicating that other metabolic adaptations in response to seasonal changes might be involved in reducing body weight, such as energy expenditure and thermogenesis.

### 4.1. Hypothalamic Regulation of Food Intake

**Figure 4:**
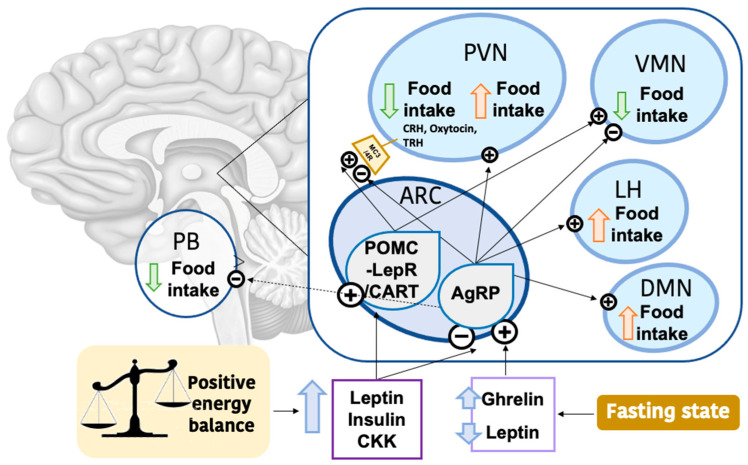
Hypothalamic regulation of food intake. When the energy balance of the organism is positive, the concentration of leptin, insulin and cholecystokinin (CKK), among others, is increased. These hormones activate the POMC/CART-expressing neurons, that upregulate the expression of anorexigenic molecules (such as orexin, Crh and TSH) in the PVN and the VMN, favoring a reduction in food intake. On the other hand, during fasting, ghrelin levels are increased and leptin levels are reduced. These changes activate the AgRP-expressing neurons, which are indeed suppressed by high leptin concentrations. This group of neurons act by stimulating food intake through the PVN, LH and DMN, while suppressing PB and VMN, preventing reduced feeding. Abbreviations: POMC: *pro-opiomelanocortin*; CART: *cocaine- and amphetamine-regulated transcript*; Crh: corticotropin-releasing hormone; TSH: thyroid-stimulating hormone; PVN: Paraventricular Nucleus; VMN: Ventromedial Nucleus; AgRP: agouti-related peptide; LH: Lateral Hypothalamus; DMN: Dorsomedial Hypothalamic Nucleus; PB: Parabranchial Nucleus.

Energy intake is in large part regulated by hypothalamic neurons, which respond to changes in nutrient and hormone levels. This includes leptin, which is sensed by leptin receptors (mainly LepRb) expressed on hypothalamic neurons [52]. Leptin mirrors the energy state in mammals: when high in circulation it inhibits energy intake, while a reduction of leptin levels favors food consumption. The arcuate nucleus of the hypothalamus (ARC) hosts the prototypical neurons responsible for regulating energy balance and responding to leptin signaling (Figure 4) [52,53]. Moreover, leptin levels also affect other regions of the brain (such as the ventromedial hypothalamus). AgRP are the ARC neurons expressing the orexigenic neurotransmitters *Agouti-related peptide* (AgRP), *Neuropeptide Y* (NPY) and *Gamma-aminobutyric Acid* (GABA) [54]. These neurons rapidly respond to changes in leptin levels, promoting food intake when levels are low. Similarly, high ghrelin concentrations stimulate feeding through AgRP activation [55]. On the other hand, high circulating levels of leptin, insulin and cholecystokinin (CCK) inhibit AgRP neurons, therefore reducing food consumption. Particularly, evidence indicates that the majority of the anti-obesity effects of leptin are mediated by the GABAergic AgRP neurons [56]. High leptin levels are also detected by neurons expressing *Pro-opiomelanocortin* (POMC), particularly with those that express the leptin receptor (LEPR), and with *cocaine-and amphetamine-regulated transcript* (CART), which, in the ARC, represent the anorexigenic group. Interestingly, CART neurons located in another region of the hypothalamus (the lateral hypothalamus) show orexigenic properties [57]. POMC-LEPR neurons directly regulate leptin levels and glucose homeostasis, but their role in reducing food intake is dependent on the feeding state rather than on leptin levels [58]. Neurons in the ARC project to other regions of the brain, inside and outside the hypothalamus (i.e., AgRP neurons mostly target the lateral parabrachial nucleus [59], while POMC neurons mostly project to the paraventricular region [55]), through the secretion of specific neurotransmitters, being able to regulate feeding behavior, among other features [55,56,58]. This machinery is complex and the exact connections that compose it are still being discovered. Nonetheless, it has been shown that changes in photoperiod can influence food intake and nutrient choices, which involves all these hypothalamic neuronal system [60].

### 4.2. Photoperiodic Regulation of Food Intake

Siberian hamsters exhibited high concentrations of leptin during LP as a response to expanded fat mass, despite no changes in food intake [61] (Table 2). This can be explained by an altered leptin sensitivity in this species [60]. This alteration was defined as the “*leptin paradox*”, a phenomenon in which hamsters become almost unsensitive to this hormone (similar to what happens in obesity) during LP, whereas leptin sensitivity strongly increased during SP. In this context, some studies reported that treating Siberian hamsters with leptin injections was more effective at reducing body mass and food intake during SP [62,63], suggesting that leptin’s ability to reduce body mass is stronger during SP than during LP. This is mechanistically explained by increased *Suppressor Of Cytokine Signaling 3* (SOCS3) expression and decreased leptin-induced *Signal transducer and activator of transcription 3* (STAT3) phosphorylation levels in the ARC during SP and food deprivation [64]. SOCS3 is part of the natural feedback loops controlling leptin signaling, and is a strong mediator of leptin and insulin resistance in obesity [65]. Interestingly, SOCS3 photoperiod-related expression changes suggest that it is altered not only during obesity, but also according to seasonality, therefore contributing to the peculiar feeding behavior. Nevertheless, the ARC may have little implications in Siberian hamsters’ seasonal changes in feeding behavior, as it has not been possible to establish any expression pattern in the main genes involved in the regulation of food intake in this region [61,63]. Additionally, lesions of the ARC following neonatal treatment with monosodium glutamate did not prevent the seasonal shift in food intake behavior [66]. Meanwhile, the expression of POMC follows its own seasonal pattern, being reduced in the ARC during SP [61,63]. Indeed, it was recently reported that POMC expression follows long-term predictive cues, as it is targeted by T3 signaling during seasonal adaptations [14]. Therefore, POMC, together with T3, could be directly involved in the photoperiodic regulation of food intake.

In F344 rats, no changes in circulating leptin concentrations between photoperiods have been reported despite the clear variations in body weight and energy intake [46,48]. However, the expression of *Corticotropin-releasing hormone* (CRH), a peptide hormone involved in the inhibition of food intake, was increased in the paraventricular nucleus of F344 rats exposed to SP compared with LP, which agrees with the feeding behavior of these animals [46]. Despite this, *Agrp* expression in the ARC was reduced while *Npy* was overregulated by SP treatment. These findings are contradictory to the feeding behavior since the combination of these two neuropeptides is usually overlapping and induces an orexigenic effect. Thus, it is likely that both molecules can be independently regulated by changes in photoperiod and develop other functions beyond regulation of food intake. In addition, neither *Cart* nor *Pomc* expression in the ARC seem to be affected by photoperiod [46,48].

### 4.3. The NMU and Wnt/β-Catenin Signaling

Alternatively, another signaling pathway that could be involved in regulating photoperiodic changes in energy balance in rodents is *Neuromedin U* (NMU). It is expressed in PT cells and has been observed to mimic the TSHβ function in F344 rats. Furthermore, it is strongly modulated by photoperiod. Particularly, NMU upregulates DIO2 expression in tanycytes, where it also regulates *Wnt/β-catenin* signaling [67]. The *Wnt/β-catenin* signaling is closely related to energy homeostasis, as it mediates leptin signaling within the ARC, showing an important role in food intake. Moreover, it seems to be involved in the control of adipose tissue functionality [68]. Similarly, some of the molecules that belong to the retinoic acid system are expressed in the same area of the hypothalamus and, again, are regulated by photoperiod. This pathway is also implicated in food intake regulation and it has been suggested to be involved in the seasonal changes in energy balance [69].

### 4.4. Photoperiodic Impact in Nutrient Choice

Seasonality might not only affect energy consumption, but it also seems to have an influence on the type of nutrients ingested (Table 3). F344 rats housed under an SP consumed less energy provided from proteins and carbohydrates than rats housed under an LP [46], while energy intake provided from fat did not change between these groups [70]. These findings suggest that nutritional requirements change depending on the season and animals are capable of selecting the type of nutrients that are more appropriate for their survival during a certain period. Interestingly, a study that assessed the effects of consuming out-of-season orange in F344 rats during SP observed altered metabolic patterns in the adipose tissue [71], suggesting that the impact of certain nutrients in the organism change depending on the season.

**Table 2:** Photoperiod-induced changes in food intake.

Species	M/F	Photoperiods (Light/Dark Hours)	Food Intake	Leptin	Hypothalamic Neuropeptides	Reference
F344 rats	M	16/8–8/16	↓ SP	↑ LP (after 8 h fasting)	Not measured	[17]
F344 rats	M	16/8–8/16	↓ SP	ND	SP ↓ *Agrp*, ↑ *Crh* ↓↓ *Pomc* in ventral ependymal region	[46]
F344 rats	M	18/6–6/18 (Control: 12/12)	↓ Cumulative food intake of SP (from week 9 onwards)	↑ LP	LP and SP ↑ *Npy* ↑ Ghrelin receptor (*Ghsr*)	[49,50]
Siberian hamsters	M	16/8–8/16	Not measured	↑ LP (iWAT and rWAT mRNA)		[72]
Siberian hamsters	M	16/8–8/16	Not measured	↑ LP	SP ↓ *Pomc,* ↓ *Obrb*, ↓ *Mc3-r*, ↑ *Cart* LP-REST ↑ *Obrb*, ↓ *Cart*	[61]
Siberian hamsters	M	16/8–8/16 Acute leptin treatment	↓ SP compared with LP ↓ SP + leptin compared to SP ↓↓ LP + leptin compared with LP		SP ↓ *Pomc* ↓ *Npy* (8 weeks) ND in *Orexin* or *Npy* (12 weeks)	[63]

Abbreviations: ARC: Arcuate Nucleus; F: female; iWAT: inguinal White Adipose Tissue; LP: Long Photoperiod; M: male; ND: No statistical differences; REST: food restriction; rWAT: retroperitoneal White Adipose Tissue; SP: Short Photoperiod; ↓: decrease; ↑: increase.

## 5. Effects of Photoperiod on Energy Expenditure

As previously mentioned, photoperiod may affect body weight not only by affecting food intake in some mammals during SP. Accordingly, an important contributor in regulating energy expenditure in rodents is the brown adipose tissue (BAT). Despite being an adipose depot, its morphology and function is completely different from the WAT. Instead of storing fat, brown adipocytes are capable of dissipating energy by inducing thermogenesis through the action of UCP1 located in the mitochondrial membrane of brown adipocytes. UCP1 enables brown adipocytes to uncouple the respiratory chain from oxidative phosphorylation and activate thermogenesis [73]. BATs functionality is required for rodents to maintain body temperature, allowing them to survive periods of nocturnal cold. BAT adipocytes are different from WAT adipocytes, with a greater number of mitochondria (which provides the typical brown color of this tissue), a multilocular content of fat droplets and high levels of innervation [74]. It has also been demonstrated that BAT is involved in regulating the clearance of TG and in glucose homeostasis, as well as its contribution as an endocrine tissue capable of releasing batokines with several effects at a systemic level [75]. When depleted from its energy supplies (mainly FA and few glycogen stores), BAT needs to obtain energy from blood (glucose and NEFAs) in order to replenish the pools and be able to oxidize it through lipolysis and glycolysis [76,77]. In this context, activation of BAT has an important role in energy metabolism, as it is capable of causing an energy imbalance [78,79]. Therefore, because of these properties, BAT activation could be a great target for obesity treatment. Unfortunately, BAT is poorly developed in adult humans. In fact, BAT content and activity decrease with aging, and even more rapidly in obese people. Nevertheless, active BAT in adult humans has recently been described, mainly in the cervical, supraclavicular, axillary and paravertebral zones [36,74]. There are also diffused brown fat cells in coexistence with WAT and skeletal muscle tissues [73].

The relevance of photoperiod to BAT activity is strongly established in mammals, with clear evidence in hibernator species. As the main photoperiodic biological signal is melatonin, its involvement in BAT functionality and browning has also been studied and demonstrated in photoperiodic and non-photoperiodic rodents, where the general findings report a BAT inducing role for melatonin [80]. Additionally in humans, it was recently reported that by treating melatonin-deficient patients with 3 mg of melatonin for 3 months, their BAT volume and activity were significantly increased [81]. Therefore, light exposure may have a direct impact in modulating energy expenditure and, consequently, body weight.

### 5.1. Photoperiodic Remodeling of BAT Metabolism

As shown in Table 4, an increase in BAT functionality was observed in Siberian hamsters exposed to winter-like photoperiod, as the expression of *Ucp1* [9,72] and *Pgc-1α* was significantly increased in this tissue [72]. Interestingly, this response was mimicked when animals were injected with melatonin, indicating that melatonin signaling might be involved in energy metabolism during seasonal adaptations [9]. Furthermore, a study performed with C57BL/6 mice reported that, under 24-h light exposure, brown adipocytes reduced its nutrient uptake from plasma [44], which agrees with previous assumptions in Siberian hamsters. In contrast, different findings have been observed in F344 rats, in which BAT activity seems to be reduced during SP. In this context, *Ucp1* was downregulated together with genes associated with thermogenesis (*Prdm16*), lipid uptake (*Cd36, Fatp1, Lpl*) and lipolysis (*Cpt1β, Hadh*). Therefore, the involvement of BAT in the energy metabolism of F344 rats might be scarce in winter-like photoperiods. Even so, previous results obtained with indirect calorimetry has revealed higher values of oxygen consumption (VO2) and a lower respiratory quotient for F344 rats exposed to SP, accompanied by increased energy expenditure and higher lipid use, respectively, for these animals [50]. Taken together, these findings suggest that SP might induce an increase in energy expenditure in F344 rats, but the mechanisms by which it occurs may not include BAT activity. This is also supported by data obtained from Collared lemmings housed under SP in which an increased total energy expenditure was observed with lower UCP1 protein expression in BAT, or in Brandt’s voles exposed to LP in which lower energy rate was detected while increased thermogenic markers in BAT were observed.

Interestingly, photoperiod-dependent differences in energy expenditure were also observed in young Wistar rats. This species is photoperiod-unsensitive, but it seems to respond to this cue during early development. Differently from F344, Wistar rats exposed to SP decreased their daily energy expenditure and resting metabolic rate [82]. It is consistent with other studies showing increased body weight in these rats during SP [83].

**Table 4:** Influence of photoperiod in BAT functionality.

Species	M/F	Photoperiods (Light/Dark Hours)	BAT Activity	Reference
F344 rats	M	18/6–6/18	SP ↓ β-oxidation-related genes ↓ FA transport ↓ Thermogenesis (not significant) ↑ Adipogenesis	[18]
C57BL/6 mice	M	12/12–16/8 (No SP group)	LP ↓ Nutrient uptake	[44]
Siberian hamsters	M	16/8–8/16	SP ↑ Thermogenesis	[9]
Siberian hamsters	M	16/8–8/16	SP ↑ Thermogenesis	[72]
Collared lemmings	M	16/8–8/16	SP ↓ Thermogenesis	[84]
Syrian Hamsters	F	16/8–8/16	SP ↑ BAT weight	[85]

Abbreviations: BAT: Brown Adipose Tissue; F: female; FA: Fatty Acid; LP: Long Photoperiod; M: male; SP: Short Photoperiod; ↓: decrease; ↑: increase.

### 5.2. Photoperiodic Regulation of Other Metabolic Tissues

Liver and muscle also exhibit photoperiodic adaptations regarding energy homeostasis. For instance, *Cd36* expression was reduced in liver and soleus muscle of F344 rats housed under SP, and β-oxidation-related genes including *Fatp1, Hadh, Cpt1β* were reduced in the soleus muscle of these animals [70]. In addition, the general metabolic activity of skeletal muscle of these rats seemed to be impaired, as increased levels of succinate, AMP, IMP and pAMPK and reduced p-Akt2 and total protein content was observed in different muscle depots. Likewise, Wistar rats that indeed gained weight during SP, showed upregulation of lipogenesis-related genes (*Pepck, Pgc1β, Fasn*) in liver, where there were higher amounts of TG and lipids but reduced total concentrations of cholesterol [83].

## 6. Impact of Obesity in the Photoperiodic Remodeling of Adiposity and Energy Metabolism

**Figure 5:**
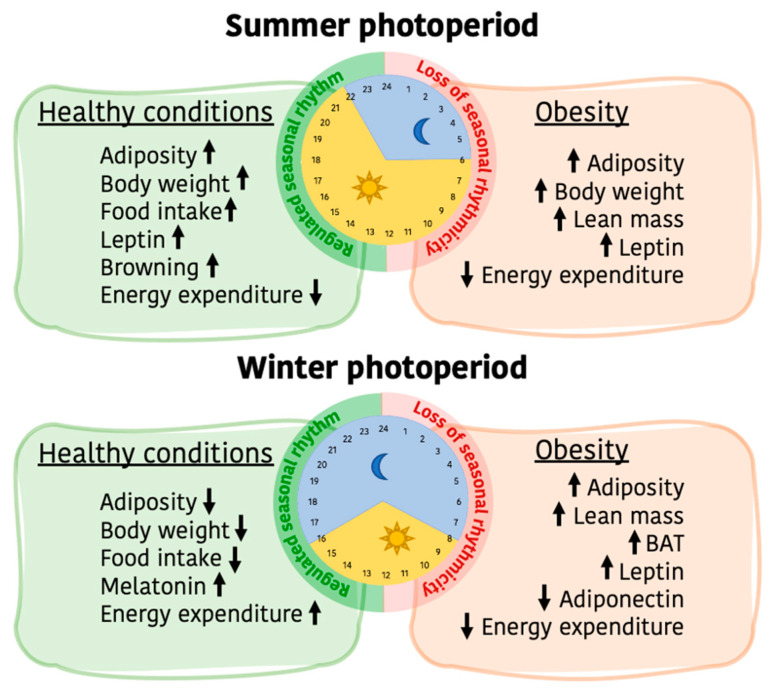
Photoperiod-dependent adaptations in energy and lipid metabolisms and the alterations caused by obesity in mammals. In healthy conditions, seasonal rhythms are regulated in mammals and adiposity, body weight, food intake, leptin signaling and browning in the adipose tissue are increased in summer photoperiod, while energy expenditure is reduced. The contrary happens in winter photoperiod, where melatonin levels are increased. However, in obesity, the biologic seasonal rhythmicity is dysregulated and energy and lipid metabolisms are altered. Anabolism is accentuated in summer photoperiod, and in winter photoperiod there is an increase in adiposity, lean mass, BAT mass and leptin signaling, while adiponectin levels are lowered and energy expenditure drops. Abbreviations: BAT: Brown Adipose Tissue.

Throughout this review, it has been demonstrated that, under a metabolically healthy state, changes in photoperiod represent important cues for body mass and adiposity adaptations, feeding behavior and energy expenditure. However, these adaptations can change when an organism faces an obesogenic environment [86], and indeed many studies have focused on investigating the possible impact of diet-induced obesity on the metabolic responses to seasonal changes (Table 5, Figure 5).

Animals fed with a high fat diet (HFD) lost their photoperiodic control of fat mass, as it was considerably increased in both LP and SP [46]. However, lean mass was maintained. Similar results were reported in genetically obese Zucker rats, in which fat mass was significantly increased in obese animals during both LP and SP, but the content of lean mass did not change between lean and obese animals during the same photoperiod. Interestingly, the mass of visceral fat was increased in lean animals exposed to LP compared with both lean and obese animals during SP [19]. Similarly, F344 rats fed with a cafeteria (CAF) diet had increased expression of genes related to adipogenesis and lipogenesis in retroperitoneal WAT (response completely opposite to that observed in non-obese animals) indicating that diet-induced obesity disrupts photoperiodic adaptations in the adipose tissue of these animals [18]. In contrast, in *Psammomys obesus* rats fed with a high energy diet (HED), the effects of photoperiod were accentuated. During SP, obese rats exhibited a greater reduction in adipogenesis-related genes in visceral and subcutaneous WAT depots, but developed larger adipocytes compared with LP. Consistently, *Pparα* levels were increased in visceral depots during SP, and *Adipoq* levels were reduced in both visceral and scWAT depots in animals exposed to the same photoperiod [41].

Concerning food intake, F344 rats fed with HFD presented a decrease in food and protein intake whereas leptin levels were increased during both photoperiods, but no subsequent changes in the ARC were detected in these animals [46]. Nevertheless, higher levels of *Npy* and *Ghsr* in the ARC were observed in both LP and SP compared with control animals. Moreover, CAF rats showed a higher preference for fat-rich food items only in LP [70]. Similarly, Sprague-Dawley rats fed with a high carbohydrate diet (HCD) presented a significant increase in sucrose consumption only when exposed to LP, and this was associated with an increase in circulating leptin levels [87].

When focusing on energy expenditure, reduced browning markers such as *Ucp1* and *Pgc-1α* were observed in WAT of *Psammomys obesus* rats fed with HED [41]. In obese Zucker rats, although BAT size was increased during LP, the mitochondrial content of this tissue was higher during SP, indicating a higher functionality [19]. In contrast, F344 rats fed with CAF diet exhibited reduced energy expenditure during both photoperiods, showing the opposite response to non-obese animals. Moreover, F344 rats exposed to SP showed reduced fat oxidation rates and increased carbohydrate oxidation rates, which was associated with a downregulation of the expression of β-oxidation-related genes including *Fatp1, Had, Cpt1β* in soleus and gastrocnemius muscle of these rats, while genes related to glucose metabolism were not modulated in this photoperiod [70].

Interestingly, when seasonal rhythms are lost, energy metabolism is affected and the risk of developing obesity is increased. This can happen when the environmental cues that provide information about photoperiod and daylength are impaired. Indeed, night-shift workers are at higher risk of developing metabolic disorders and therefore becoming overweight and obese. Hence, even if these metabolic abnormalities are often related to the disruption of circadian rhythms rather than circannual cycles, it has been demonstrated that if there is a longer duration of working night-shift, the risk of developing alterations in metabolism such as type 2 diabetes is higher [88]. In this context, the influence of light at night may be of significance, as studies with rodents reported that exposure to dim light at night not only caused body weight gain in mice, but also glucose intolerance in rats [89,90]. Additionally, it was shown that women exposed to artificial light at night while sleeping increased their risk of developing obesity [91], and previous studies in men showed that responses to seasonal changes in the secretion of hormones such as melatonin, cortisol or thyrotropin, were lost when subjects were exposed to artificial lighting [92].

Another important system of the organism that is affected by seasonal changes is the gut microbiota. Moreover, the gut microbiota rapidly responds to an obesogenic environment, where its composition is altered. A recent study revealed that the impact of photoperiod in fecal bacteria was accentuated in cafeteria-fed rats [93]. In fact, the obesity-derived alterations in the fecal microbiota were exaggerated in animals housed under long photoperiod conditions [93].

Altogether, these findings demonstrate an important impact of obesity in disrupting photoperiodic adaptations. To prevent the alterations induced by obesity and therefore reduce the metabolic abnormalities associated with this disease, it would be interesting to consider the known natural responses shown by the metabolic tissues involved in seasonal adaptations to find strategies that help maintain the seasonal rhythm of these processes.

## 7. Concluding Remarks

- Research on seasonal rhythms and associated physiological responses goes back to the past century, but there is still a wide gap in the current understanding of its impact on humans. Important advances in knowledge have been achieved to date, such as describing and establishing the basic mechanisms by which seasonal changes are received and expanded through the organism. Animal-based studies have shown that the main seasonal cue is daylength, and metabolic adaptations are developed to guarantee species survival.
- These metabolic adaptations naturally involve changes in body weight, which are mainly based on adiposity. Here, we have highlighted the seasonal modifications reported in WAT, showing that the impact of photoperiod in this tissue is noticeable. However, the specific metabolic pathways still need to be drawn in order to understand the impact of seasonality on WAT functionality. WAT is an important metabolic tissue whose function is highly affected by obesity. In the situation of obesity, seasonal adaptations in adipose are not possible and the organism is less protected against environmental adversities.
- Changes in food intake are also related to photoperiod, involving both the amount of energy consumed and the type of nutrients ingested. Evidence suggests that seasonal food intake regulation is set in different regions of the hypothalamus, where molecules other than the already established ones could have an important role. In this context, further studies that explore the role of these specific hypothalamic regions and molecules would help decipher the full mechanisms by which food intake is seasonally regulated. Considering that seasonal regulation of food intake is altered by obesity, as well as by disturbed environmental cues which can lead to wrong responses in food consumption, it is important to maintain seasonal patterns in feeding behavior.
- BAT activity and browning are deeply involved in regulating energy expenditure, and are induced by melatonin signaling that follows changes in photoperiod. Knowing that human BAT also follows this pattern, it would be interesting to promote this feedback mechanism through favoring already known methods or new strategies that boost melatonin production.
- Overall, this review has demonstrated that changes in photoperiod have an important regulatory role in energy balance and metabolism. Molecular changes in the main metabolic tissues have been observed and are described here, providing new perspectives in the regulation of energy metabolism. Applied research in seasonal animals and humans will possibly help understanding the long-term regulation of food intake and body weight.

## Figures and Tables

**Table 1:** Influence of photoperiod in body weight, adiposity and WAT metabolism.

Species	M/F	Photoperiods (Light/Dark Hours)	Body Weight	Adiposity	WAT Metabolism	Reference
F344 rats	M	18/6–6/18	=	↓ SP	SP ↓ Hypertrophy ↑ Hyperplasia ↓ Adipogenesis ↓ Lipogenesis ↓ Browning	[18]
Siberian hamsters	M	16/8–8/16	↓ SP	↓ SP	SP ↑ Lipolysis	[9]
Field voles	M	16/8–8/16	↓ SP	↓ SP	SP = Lipogenesis	[43]
Brandt voles	M	16/8–8/16	↓ SP	↓ SP	SP ↓ Lipogenesis ↓ Hypertrophy LP ↑ Browning	[42]
*Psammomys obesus* rats	M	12/12–5/19	=	=	SP ↓ Adipogenesis ↓ Browning	[41]
C57BL/6 mice	M	12/12–16/8	↑ LP	↑ LP	LP ↓ Glucose uptake	[44]

Abbreviations: LP: long photoperiod; SP: short photoperiod; WAT: white adipose tissue; ↓: decrease; ↑: increase; =: no changes observed.

**Table 3:** Photoperiod-dependent nutrient choices.

Species	M/F	Photoperiods (Light/Dark Hours)	Carbohydrates	Proteins	Fat	Reference
F344 rats	M	16/8–8/16	↑ LP	↑ LP	ND	[17]
F344 rats	M	16/8–8/16	Not measured	↑ LP	Not measured	[46]
F344 rats	M	18/6–6/18 (Control: 12/12)	↓ LP and SP	Not measured	ND	[49,50]

Abbreviations: F: female; LP: Long Photoperiod; M: male; ND: No statistical Differences; SP: Short Photoperiod; ↓: decrease; ↑: increase.

**Table 5:** Influence of obesity in photoperiod adaptations.

Species	M/F	Photoperiods (Light/Dark Hours) and Diet	Adiposity	Food Intake	Energy Expenditure	Reference
F344 rats	M	18/6–6/18 STD or CAF	LP-CAF ↓ Lean/fat mass ratio	SP ↓ Cumulative food intake LP-CAF ↑ Preference for fat-rich foods SP-CAF and LP-CAF ↓ CH Consumption and ↑ *Npy* ↑ *Ghsr*	SP-CAF ↑ RQ → ↑ CH oxidation ↓ fat oxidation rates SP-CAF and LP-CAF ↓ EE	[50,70]
F344 rats	M	18/6–6/18 STD or CAF	SP-STD and SP-CAF ↑ Number of smaller adipocytes SP-CAF ↑ *Acacα, C/ebpα* and FASN	No data	SP-CAF ↑ BAT (%) ↑ lean mass (%)	[18]
F344 rats	M	16/8–8/16 STD or CAF	SP-CAF and LP-CAF Doubled adiposity ↑ TG, NEFAs	SP-HFD and LP-HFD ↓ Food and protein intake and ↑ Leptin SP-HFD ↓↓ *AgRP* and *Pomc*	LP-STD and LP-CAF ↑ Lean mass	[46]
*Psammomys* *obesus* rats	M	12/12–5/19 LED or HED	SP-LED and SP-HED ↑ Larger adipocytes SP-HED Larger adipocytes and ↑ *Pparα* ↓↓ *Pparγ*, *C/ebpα* and *Adipoq* SP-HED and LP-HED ↓ *Pparγ* (visceral WAT)	No data	SP-HED ↓↓ *Pgc-1α* (scWAT) → ↓ browning ↑ *Ucp1* (visceral WAT)	[41]
Zucker rats	M	14/10–10/14 Genetically obese or lean	LP-Lean ↑ Visceral WAT LP-Obese ↑↑ total fat	SP-Obese and LP-Obese ↑↑ Food consumption	LP-Obese ↑ BAT SP-Obese ↓ lean mass gain SP-Lean and SP-Obese ↑↑ mitochondrial content	[19]

Abbreviations: BAT: Brown Adipose Tissue; CAF: cafeteria; CH: Carbohydrate; EE: Energy Expenditure; F: female; HED/LED: High/Low Energy Diet; LP: Long Photoperiod; M: male; NEFAs: Non-Esterified Fatty Acids; RQ: Respiratory Quotient; SP: Short Photoperiod; STD: Standard; TG: Triglycerides; ↓: decrease; ↑: increase.
